# Comparative Effectiveness of Carotid Endarterectomy vs Initial Medical Therapy in Patients With Asymptomatic Carotid Stenosis

**DOI:** 10.1001/jamaneurol.2020.1427

**Published:** 2020-06-01

**Authors:** Salomeh Keyhani, Eric M. Cheng, Katherine J. Hoggatt, Peter C. Austin, Erin Madden, Paul L. Hebert, Ethan A. Halm, Ayman Naseri, Jason M. Johanning, Danielle Mowery, Wendy W. Chapman, Dawn M. Bravata

**Affiliations:** 1 Division of General Internal Medicine, Department of Medicine, University of California San Francisco, San Francisco; 2 San Francisco Veterans Affairs (VA) Medical Center, San Francisco, California; 3 Department of Neurology, UCLA (University of California Los Angeles), Los Angeles; 4 VA Greater Los Angeles Healthcare System, Los Angeles, California; 5 Institute of Health Policy, Management and Evaluation, University of Toronto, Toronto, Ontario, Canada; 6 Northern California Institute of Research and Education, San Francisco; 7 University of Washington, Seattle; 8 Puget Sound VA, Seattle, Washington; 9 Department of Internal Medicine, University of Texas Southwestern Medical Center, Dallas; 10 Department of Population, University of Texas Southwestern Medical Center, Dallas; 11 Department of Data Science, University of Texas Southwestern Medical Center, Dallas; 12 Department of Ophthalmology, University of California San Francisco, San Francisco; 13 Department of Surgery, University of Nebraska, Omaha; 14 Omaha VA Medical Center, Omaha, Nebraska; 15 Biomedical Informatics, University of Utah, Salt Lake City; 16 Salt Lake City VA Health Care System, Salt Lake City, Utah; 17 Now with Department of Biostatistics, Epidemiology, & Informatics, University of Pennsylvania, Philadelphia; 18 University of Melbourne, Melbourne, Victoria, Australia; 19 Department of Medicine, Indiana University School of Medicine, Indianapolis; 20 Department of Neurology, Indiana University School of Medicine, Indianapolis; 21 Richard L. Roudebush VA Medical Center, Indianapolis, Indiana

## Abstract

**Importance:**

Carotid endarterectomy (CEA) among asymptomatic patients involves a trade-off between a higher short-term perioperative risk in exchange for a lower long-term risk of stroke. The clinical benefit observed in randomized clinical trials (RCTs) may not extend to real-world practice.

**Objective:**

To examine whether early intervention (CEA) was superior to initial medical therapy in real-world practice in preventing fatal and nonfatal strokes among patients with asymptomatic carotid stenosis.

**Design, Setting, and Participants:**

This comparative effectiveness study was conducted from August 28, 2018, to March 2, 2020, using the Corporate Data Warehouse, Suicide Data Repository, and other databases of the US Department of Veterans Affairs. Data analyzed were those of veterans of the US Armed Forces aged 65 years or older who received carotid imaging between January 1, 2005, and December 31, 2009. Patients without a carotid imaging report, those with carotid stenosis of less than 50% or hemodynamically insignificant stenosis, and those with a history of stroke or transient ischemic attack in the 6 months before index imaging were excluded. A cohort of patients who received initial medical therapy and a cohort of similar patients who received CEA were constructed and followed up for 5 years. The target trial method was used to compute weighted Kaplan-Meier curves and estimate the risk of fatal and nonfatal strokes in each cohort in the pragmatic sample across 5 years of follow-up. This analysis was repeated after restricting the sample to patients who met RCT inclusion criteria. Cumulative incidence functions for fatal and nonfatal strokes were estimated, accounting for nonstroke deaths as competing risks in both the pragmatic and RCT-like samples.

**Exposures:**

Receipt of CEA vs initial medical therapy.

**Main Outcomes and Measures:**

Fatal and nonfatal strokes.

**Results:**

Of the total 5221 patients, 2712 (51.9%; mean [SD] age, 73.6 [6.0] years; 2678 men [98.8%]) received CEA and 2509 (48.1%; mean [SD] age, 73.6 [6.0] years; 2479 men [98.8%]) received initial medical therapy within 1 year after the index carotid imaging. The observed rate of stroke or death (perioperative complications) within 30 days in the CEA cohort was 2.5% (95% CI, 2.0%-3.1%). The 5-year risk of fatal and nonfatal strokes was lower among patients randomized to CEA compared with patients randomized to initial medical therapy (5.6% vs 7.8%; risk difference, −2.3%; 95% CI, −4.0% to −0.3%). In an analysis that incorporated the competing risk of death, the risk difference between the 2 cohorts was lower and not statistically significant (risk difference, −0.8%; 95% CI, −2.1% to 0.5%). Among patients who met RCT inclusion criteria, the 5-year risk of fatal and nonfatal strokes was 5.5% (95% CI, 4.5%-6.5%) among patients randomized to CEA and was 7.6% (95% CI, 5.7%-9.5%) among those randomized to initial medical therapy (risk difference, −2.1%; 95% CI, −4.4% to −0.2%). Accounting for competing risks resulted in a risk difference of −0.9% (95% CI, −2.9% to 0.7%) that was not statistically significant.

**Conclusions and Relevance:**

This study found that the absolute reduction in the risk of fatal and nonfatal strokes associated with early CEA was less than half the risk difference in trials from 20 years ago and was no longer statistically significant when the competing risk of nonstroke deaths was accounted for in the analysis. Given the nonnegligible perioperative 30-day risks and the improvements in stroke prevention, medical therapy may be an acceptable therapeutic strategy.

## Key Points

**Question:**

Among patients with asymptomatic carotid stenosis, is carotid endarterectomy superior to initial medical therapy in preventing fatal and nonfatal stroke within 5 years of follow-up in real-world practice?

**Findings:**

In this comparative effectiveness study of 5221 patients with asymptomatic carotid stenosis, the absolute reduction in the risk of fatal and nonfatal strokes associated with early carotid endarterectomy treatment was less than half the reduction observed in trials initiated more than 2 decades ago. The decrease was not statistically significant when the competing risk of nonstroke deaths was accounted for in the analysis.

**Meaning:**

Results of this study suggest that, given the up-front perioperative risks associated with carotid endarterectomy and the reduced benefit derived from revascularization, initial medical therapy may be an acceptable treatment strategy for the management of asymptomatic carotid stenosis.

## Teaser

This comparative effectiveness study describes the analytical strategy, methods, and results of a target trial emulation examining cohorts of US veterans with carotid stenosis.

## Introduction

Randomized clinical trials (RCTs) have established that carotid endarterectomy (CEA) is beneficial in preventing stroke in both asymptomatic^1,2,3^ and symptomatic patients^4,5^ with carotid stenosis. However, the most recent of the 3 trials that established the clinical benefit of CEA compared with medical therapy in asymptomatic patients was initiated 25 years ago.^1,2,3^ In the intervening period, new pharmacological advances, such as high-potency statins^6,7,8,9^ and improved antiplatelet regimens,^10,11^ as well as quality improvements in the treatment of blood pressure^12,13^ and diabetes^14^ may be associated with the reduced stroke rate among patients with asymptomatic carotid stenosis. Empirical evidence in support of a reduction in stroke risk among patients with asymptomatic carotid stenosis has been provided by a systematic review of several studies.^15^ If the decrease in stroke rate among patients with carotid artery stenosis is associated with improvements in primary stroke prevention, revascularization may no longer be the preferred treatment strategy.

Since the first RCT was published that demonstrated the superiority of CEA compared with medical therapy for patients with asymptomatic carotid stenosis, concerns have emerged that the outcomes seen in a clinical trial setting may not be reproducible in non-RCT settings because of demonstrated less optimal surgical outcomes,^16,17,18,19^ higher complication rates in low-volume hospitals,^20,21,22^ and poor patient selection.^23,24^ The risk to benefit ratio of interventions may be less favorable outside of RCTs. In addition, although a much larger clinical benefit has been observed in treating patients with symptomatic carotid stenosis, most interventions have been performed on patients with asymptomatic carotid stenosis.^25,26,27,28^ Furthermore, substantial international variation exists in using revascularization for the treatment of asymptomatic carotid stenosis, suggesting changing attitudes about the use of CEA. These changing practice patterns indicate a need to improve understanding of the long-term clinical benefit of this procedure outside of an RCT.

In this study, we used data from the US Department of Veterans Affairs (VA) and Medicare to mimic the design and analysis methods of an RCT and to examine whether early intervention (CEA) was superior to initial medical therapy in real-world practice. We hypothesized that the clinical superiority once observed in patients who received CEA has diminished, making medical therapy a more favorable treatment strategy in comparison. We also hypothesized that among carefully selected patients, CEA may still be superior to medical therapy.

## Methods

The institutional review board of the University of California San Francisco approved this study and waived the need for patient consent because the research involved no more than minimal risk to participants. This study was conducted from August 28, 2018, to March 2, 2020. We followed the International Society for Pharmacoeconomics and Outcomes Research (ISPOR) reporting guideline.

### Study Population, Data Sources, and Preliminary Screening

Using VA national data, we identified 219 979 veterans of the US Armed Forces aged 65 years or older with at least 1 visit to a VA facility for primary care (eg, general medicine, women clinic) or subspecialty care (eg, geriatrics, cardiology, endocrine, pulmonary, or neurology) who received diagnostic carotid imaging (ie, carotid ultrasonography, computed tomography angiography, or magnetic resonance angiography) between January 1, 2005, and December 31, 2009, as indicated by *Current Procedural Terminology* (*CPT*) and *International Classification of Diseases, Ninth Revision* (*ICD-9*) codes (eTable 1 in the Supplement). To create a cohort of patients with asymptomatic carotid stenosis, we excluded all patients with carotid imaging test results who had any form of stroke or transient ischemic attack (TIA) in the 6 months before the imaging test using a highly sensitive algorithm based on *ICD-9* codes^29^; we modified the algorithm for this study to also exclude retinal strokes and TIAs (eTable 2 in the Supplement).

All patient demographic information, administrative data (eg, comorbid conditions, utilization), vital signs, laboratory data, and pharmacy data were retrieved from the VA Corporate Data Warehouse and Medicare data.^30^ Text clinical notes for carotid image reports were identified from the VA Corporate Data Warehouse. Data on carotid revascularization were retrieved from the VA Corporate Data Warehouse using the *ICD-9* code for CEA (38.12), *ICD-9* codes for carotid artery stenting (CAS) (00.61 and 00.63), *CPT* code for CEA (35301), and *CPT* codes for CAS (37215 and 37216). Death and cause of death were obtained from the Suicide Data Repository, which is jointly administered by the US Department of Defense and the VA. The Suicide Data Repository, created to track deaths by suicide among military personnel, contains comprehensive cause-of-death data for all US veterans.^31^

**Figure 1.**
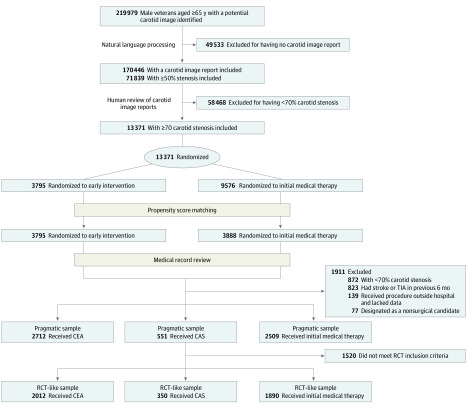
Carotid Cohort Construction CAS indicates carotid artery stenting; CEA, carotid endarterectomy; RCT, randomized clinical trial; and TIA, transient ischemic attack.

Using a previously developed natural language processing algorithm,^32^ we excluded all carotid imaging results showing stenosis of less than 50% or hemodynamically insignificant stenosis (Figure 1). We imported all text reports for patients with at least 1 carotid imaging showing stenosis of 50% or greater (n = 71 839) into a database for human screening. In this preliminary screening process, we identified all patients who had carotid imaging text reports indicating greater than 70% carotid stenosis or a qualitative description of near occlusion, critical stenosis, or severe stenosis. Subsequent medical record review was used to confirm stenosis. We identified 13 371 potentially eligible patients with stenosis of 70% or greater (Figure 1).

### Patient Matching 

Administrative data on baseline characteristics demonstrated that patients who received CEA were different from those who received initial medical therapy. For example, patients in the CEA cohort compared with patients in the initial medical therapy cohort were younger and experienced less comorbidity (eg, coronary artery disease, chronic obstructive pulmonary disease, and chronic kidney disease) (eTable 3 in the Supplement).

The first step in our analytical strategy was to construct a sample of patients in the initial medical therapy cohort who were similar to those in the CEA cohort. We used propensity score matching to identify these similar patients. Specifically, we fit a generalized boosted model for treatment (CEA vs initial medical therapy) as a function of covariates.^33^ Using the estimated propensity scores, we used a nearest neighbor match (with a caliper that varied between 0.2 and 0.8) to select patients from the initial medical therapy cohort who were similar to patients in the CEA cohort on the basis of measured covariates. At this stage, we retained all patients matched according to measured covariates because we expected some patients might be excluded during the medical record review phase.

This first step in the analytical strategy created a cohort of potentially eligible patients who were similar in measured baseline covariates. To examine the degree to which this patient matching was successful, we examined the risk of pneumonia (a tracer outcome) between the 2 matched samples (CEA vs initial medical therapy) within 1 year of the index carotid imaging. We found no difference in the risk of pneumonia, suggesting that matching was successful.

### Medical Record Review

Next, we reviewed the medical record of each potentially eligible patient to confirm both carotid stenosis level and symptom status (hemorrhagic or ischemic stroke or TIA in the 6 months before index imaging). We excluded patients with a history of stroke or TIA in the 6 months before index imaging. Patients with carotid stenosis levels between 50% and 70% were also excluded because recent trials have included patients with a higher threshold of stenosis, and practice patterns have moved toward revascularization of patients with greater stenosis who are perceived at higher risk of stroke.^34^ We also collected from the medical record data on disease severity (eg, ejection fraction, pulmonary function) that were not available in national VA data; data on receipt of aspirin at baseline because aspirin is an over-the-counter medication that may not be dispensed by VA pharmacies; and data on whether the patient underwent a CEA or CAS and whether the patient had a history of remote stroke or TIA more than 6 months before index carotid imaging. Details on the abstraction and quality assurance protocols during the medical abstraction phase are provided in eTable 4 in the Supplement.

Among the 3795 patients designated as the CEA cohort and the 3888 propensity-matched patients in the initial medical therapy cohort, 823 patients were excluded because of a previous stroke or TIA and 872 patients were excluded because they did not meet stenosis criteria. Also excluded were 139 patients with an ambiguous group classification because the medical record review suggested they had received a procedure from an outside hospital; however, we were unable to verify the procedure in the VA or Medicare databases. Moreover, we excluded 77 patients from the initial medical therapy cohort when a clinician specifically documented in the medical record that the patient was not a surgical candidate.

After application of the exclusion criteria, the total cohort comprised 2712 patients who received CEA, 551 who received CAS, and 2509 who received initial medical therapy (Figure 1). Because too few patients who received CAS were included, this study focused on the comparative effectiveness of CEA vs initial medical therapy, and patients who underwent CAS were excluded from all subsequent analyses.

### Emulation of Previous Trials

We emulated the analyses used in the Asymptomatic Carotid Surgery Trial (ACST),^3^ the last published RCT to estimate the comparative effectiveness of CEA vs initial medical therapy in preventing fatal and nonfatal strokes. The analytical plan for the present trial emulation was to mimic not only the ACST but also clinical practice in that a clinician might choose initial medical therapy for a patient but may later adopt a more aggressive course, such as CEA, if the patient’s condition or carotid stenosis worsens. Because of delays in receiving treatment after carotid imaging and the possibility that patients in the initial medical therapy cohort may develop an indication (ie, stroke or TIA) to switch to CEA or CAS,^3^ we, as in the ACST, operationally defined the intervention cohort as follows: receipt of intervention (CEA) within the first year after carotid imaging. We also defined the initial medical therapy cohort as follows, similar to that in the ACST: absence of intervention in the year after index carotid imaging (initial medical therapy). Thus, the target trial was of early intervention (within 1 year) vs initial medical therapy (≥1 year after).

We constructed 2 alternative samples to emulate 2 variations in trials. First, we emulated an RCT with few inclusion criteria (ie, a pragmatic RCT reflecting real-world practice). Second, we emulated a trial that mimicked the strict inclusion and exclusion criteria of carotid intervention trials.^3,35,36,37^ For the pragmatic RCT emulation, all eligible asymptomatic patients were included in the analyses (pragmatic sample), whereas for the strict criteria emulation, the analytical sample was restricted to those who met carotid trial inclusion and exclusion criteria (RCT-like sample) (eTable 5 in the Supplement).^3,34,36^

### Outcome Measure and Follow-up

We considered time zero (baseline) to be the time of the first carotid image demonstrating stenosis. As in the ACST, the combined end point of fatal and nonfatal strokes (all territorial strokes) was chosen as the primary outcome. Stroke that occurred during the follow-up period was assessed using the Tirschwell high-specificity algorithm.^38^

All patients were followed up for as long as 5 years from the date of the first carotid image exhibiting stenosis of 70% or greater. We were unable to incorporate into the analysis the perioperative complication of death associated with CEA. Unlike in a trial in which randomization to surgical intervention occurs quite rapidly, in real-world practice the delay between index carotid imaging and receipt of CEA (treatment randomization) may be longer. Therefore, we were unable to create a meaningful time frame for examining the comparable 30-day risk of death in the initial medical therapy cohort.

### Statistical Analysis

In each of the 2 analytic samples (pragmatic and RCT-like), we conducted 3 different statistical analyses: (1) estimation of crude 5-year stroke risk, (2) emulation of the ACST using target trial methods, and (3) emulation of the ACST using target trial methods and accounting for the competing risk of death. A total of 6 analyses were performed.

First, we computed Kaplan-Meier estimates of a 5-year stroke risk from the date of index imaging to allow comparisons of fatal and nonfatal stroke risk in this sample to risk in other populations. Second, we used an analytical approach, the target trial method, that allows for the assessment of observational data in a manner that emulates the analysis of an RCT in which the active intervention can be applied at varying times after baseline.^39,40,41^ To minimize the immortal time bias that can arise in an RCT when the intervention may be received some time after baseline, we randomized each patient at baseline to one of the 2 treatment cohorts (CEA or initial medical therapy), possibly contrary to the treatment that the patient actually received (see the Discussion for further detail on the target trial method).

Because this study was not truly an RCT, patients whose actual treatment did not match the randomized treatment were censored on the date they received their actual treatment (ie, the date on which the patient diverged from the randomized treatment). Patients were followed up after baseline using the observational data until the first of 4 events occurred: (1) fatal or nonfatal stroke (outcome), (2) end of the study period (administrative censoring 5 years after baseline), (3) treatment status became inconsistent with the randomized treatment status (treatment switching), or (4) enrollment in Medicare Advantage (health maintenance organization) between baseline and 1 year (censored because procedures and diagnoses recorded under those plans were not available).^42^ Thus, patients who were randomized to the initial medical therapy cohort and who subsequently underwent CEA within 1 year would be censored because of treatment switching on the date they underwent CEA; once they underwent CEA (within 1 year), their current treatment was no longer consistent with the cohort to which they were randomized (initial medical therapy). Similarly, patients who were randomized to the CEA cohort and who had not undergone CEA within 1 year would be censored after 1 year (because their treatment at 1 year was, at that point, no longer consistent with the cohort to which they were randomized). Patients who received concurrent CAS and CEA, coronary artery bypass graft concurrent with CEA, or aortic valve replacement concurrent with CEA were also censored because their treatment was not consistent with the cohort to which they were randomized. We considered a concurrent procedure to be one that occurred within 1 day of the CEA.

Because the actual choice of treatment (CEA vs initial medical therapy) may have depended on patients’ baseline and postbaseline risk and prognostic factors, we accounted for informative censoring due to treatment switching by incorporating inverse probability of censoring weights (IPCW) in the analysis. We estimated the IPCW using a Cox proportional hazards regression model that included measured baseline (time invariant) and time-varying covariates (the full list of variables included in the censoring model is provided in eTable 6 in the Supplement). We then fit a Cox proportional hazards regression model for the outcome (fatal and nonfatal strokes) as a function of an indicator variable for the randomized treatment cohort (CEA vs initial medical therapy) and incorporated the time-varying IPCW. Similar to what was observed in the ACST, regression diagnostics in this study revealed violations of the proportional hazards regression assumption (including crossing CEA and initial medical therapy survival curves); thus, we focused on estimating and reporting 5-year fatal and nonfatal stroke risk. Specifically, we estimated the 5-year risk of fatal and nonfatal strokes in the CEA and initial medical therapy cohorts incorporating the IPCW, and then we computed the risk difference by comparing the CEA cohort with the initial medical therapy cohort. We computed the end point and 95% CI estimate of the 5-year risk and risk difference from the 50th, 2.5th, and 97.5th percentiles of the bootstrap distribution for the risks and risk difference.

Third, since the publication of the ACST in 2004,^3^ accounting for competing risks has become standard in survival analyses. To reflect current statistical practice in survival analysis, we accounted for competing risks (deaths due to causes other than stroke) and repeated the target trial analyses described earlier.^43^ To do so, we estimated the cumulative incidence functions for fatal and nonfatal strokes in the CEA and initial medical therapy cohorts, accounting for nonstroke deaths as competing risks. These cumulative incidence functions were weighted by the time-varying IPCW.

Statistical significance at the α = .05 level was inferred on the basis of the bootstrapped CI for the cumulative incidence function curves. Analyses were performed using SAS Enterprise Guide, version 7.1 (SAS Institute Inc) and R, version 3.6.1 (R Foundation for Statistical Computing).

## Results

### Baseline Characteristics

Of the total 5221 patients, 2712 (51.9%; mean [SD] age, 73.6 [6.0] years; 2678 men [98.8%]) received CEA and 2509 (48.1%; mean [SD] age, 73.6 [6.0] years; 2479 men [98.8%]) received initial medical therapy within 1 year after the index carotid imaging. Table 1 provides baseline characteristics of the propensity-matched sample comparing patients in the CEA cohort with those in the initial medical therapy cohort. The baseline sociodemographic characteristics of the patients were similar, with no difference in mean age, race/ethnicity, marital status, and Medicaid enrollment. Standardized differences among 58 baseline variables were less than 0.1 except in 2 variables (remote stroke and enrollment in Medicare Managed Care). The prevalence of remote stroke was less common among the CEA cohort compared with the initial medical therapy cohort (22.0% [n = 597] vs 27.1% [n = 681]; standardized difference, −0.11). In addition, patients in the CEA cohort were less commonly enrolled in Medicare managed care at baseline (283 [10.4%] vs 406 [16.2%]; standardized difference, −0.17) (Table 1).

**Table 1.** Baseline Characteristics of Patients in Both Treatment Cohorts

Characteristic	No. (%)		Standardized difference
	CEA cohort (n = 2712)	Initial medical therapy cohort (n = 2509)	
Age, mean (SD), y	73.6 (6.0)	73.6 (6.0)	−0.043
Race/ethnicity			
White	2577 (95.0)	2368 (94.4)	0.029
Black	107 (4.0)	111 (4.4)	−0.024
Other	28 (1.0)	30 (1.2)	–0.016
Hispanic	94 (3.5)	93 (3.7)	–0.013
Men	2678 (98.8)	2479 (98.8)	–0.005
Married	1594 (58.8)	1460 (58.2)	
Veteran priority score			
High	2178 (80.3)	2013 (80.2)	0.002
Low	509 (18.8)	463 (18.5)	0.008
Unknown/missing	25 (0.9)	33 (1.3)	–0.037
Enrolled in Medicaid	133 (4.9)	111 (4.4)	0.023
Enrolled in Medicare HMO	283 (10.4)	406 (16.2)	–0.170
Comorbid conditions			
Hypertension	2400 (88.5)	2224 (88.6)	–0.005
Hyperlipidemia	2352 (86.7)	2187 (87.2)	–0.013
Diabetes	1061 (39.1)	985 (39.3)	–0.003
Ischemic heart disease	1264 (46.6)	1146 (45.7)	0.019
Remote stroke or TIA (>6 mo prior)	597 (22.0)	681 (27.1)	–0.119
Hemiplegia or other paralytic syndrome	51 (1.9)	51 (2.0)	–0.011
Peripheral vascular disease	655 (24.2)	630 (25.1)	–0.022
Abdominal aortic aneurysm	146 (5.4)	143 (5.7)	–0.014
Atrial fibrillation	258 (9.5)	190 (7.6)	–0.070
Arrhythmia other than atrial fibrillation	148 (5.5)	128 (5.1)	0.016
Valvular heart disease	206 (7.6)	174 (6.9)	0.026
DVT or PE	25 (0.9)	19 (0.8)	0.018
Hepatitis	32 (1.2)	19 (0.8)	0.043
Rheumatoid arthritis	31 (1.1)	29 (1.2)	–0.001
Pulmonary fibrosis	14 (0.5)	17 (0.7)	–0.021
Prostate cancer	158 (5.8)	111 (4.4)	0.064
Congestive heart failure			
Severe (EF <35%)	110 (4.1)	118 (4.7)	–0.032
Mild (EF ≥35%)	1218 (44.9)	1123 (44.8)	0.003
Missing EF	37 (1.4)	29 (1.2)	0.006
No congestive heart failure	1347 (49.7)	1239 (49.4)	0.006
Chronic obstructive pulmonary disease			
Moderate/severe	211 (7.8)	219 (8.7)	–0.035
Mild	227 (8.4)	192 (7.7)	0.026
Unknown severity	229 (8.4)	208 (8.3)	0.006
No chronic obstructive pulmonary disease	2045 (75.4)	1890 (75.3)	0.002
Chronic kidney disease			
Severe (GFR <30)	160 (5.9)	149 (5.9)	–0.002
Mild (GFR ≥30)	1104 (40.7)	1026 (40.9)	–0.004
No chronic kidney disease	1448 (53.4)	1334 (53.2)	0.005
Vital signs			
Systolic BP			
Normal (<120)	427 (15.7)	391 (15.6)	0.004
Prehypertension (120-139)	1271 (46.9)	1142 (45.5)	0.027
High BP stage 1 (140-159)	663 (24.5)	657 (26.2)	–0.04
High BP stage 2 (≥160)	338 (12.5)	303 (12.1)	0.012
Unknown	13 (0.5)	16 (0.6)	–0.021
Diastolic BP			
Normal (<80)	2130 (78.5)	1983 (79.0)	–0.012
Prehypertension (80-89)	437 (16.1)	414 (16.5)	–0.011
High BP stage 1 (90-99)	113 (4.2)	83 (3.3)	0.045
High BP stage 2 (≥100)	19 (0.7)	13 (0.5)	0.023
Unknown	13 (0.5)	16 (0.6)	–0.212
Body mass index			
Underweight	24 (0.9)	24 (1.0)	–0.008
Normal or healthy weight	645 (23.8)	639 (25.5)	–0.039
Overweight	1203 (44.4)	1094 (43.6)	0.015
Obese	810 (29.9)	715 (28.5)	0.031
Unknown	30 (1.1)	37 (1.5)	–0.033
Procedure			
Defibrillator	18 (0.7)	27 (1.1)	–0.044
Pacemaker	40 (1.5)	36 (1.4)	0.003
Mental health in past year			
Posttraumatic stress disorder	73 (2.7)	60 (2.4)	0.019
Depression	26 (1.0)	14 (0.6)	0.046
Anxiety	78 (2.9)	58 (2.3)	0.036
Psychosis	84 (3.1)	72 (2.9)	0.013
Social and behavioral risk factors			
Current smoker	855 (31.5)	827 (33.0)	–0.031
Alcohol abuse in past year	204 (7.5)	178 (7.1)	0.016
Drug abuse in past year	32 (1.2)	31 (1.2)	–0.005
Utilization			
≥1 Intensive care unit visit			0.071
≥1 Hospital admission	520 (19.2)	426 (17.0)	0.057
Functional status			
Nursing home in past year	17 (0.6)	21 (0.8)	–0.025
Medication			
Antiplatelet	2322 (85.6)	2129 (84.9)	0.022
Antiarrhythmic	39 (1.4)	32 (1.3)	0.014
Corticosteroid	233 (8.6)	204 (8.1)	0.017
Benzodiazepine	232 (8.6)	250 (10.0)	–0.049
Opioid	181 (6.7)	152 (6.1)	0.025
Antianginal	519 (19.1)	543 (21.6)	–0.062
Disease-modifying antirheumatic drug	33 (1.2)	25 (1.0)	0.021
Anticoagulant	27 (1.0)	22 (0.9)	0.012
Adherence			
Adherence to antihypertension medication			
Yes	1839 (67.8)	1725 (68.8)	–0.020
No	561 (20.7)	499 (19.9)	0.020
Not hypertensive	312 (11.5)	285 (11.4)	0.005
Adherence to statin therapy			
Yes	1255 (46.3)	1184 (47.2)	–0.018
No	819 (30.2)	765 (30.5)	–0.006
Not receiving statin therapy	638 (23.5)	560 (22.3)	0.029
Trial exclusion			
Acute myocardial infarction in past 30 d	25 (0.9)	18 (0.7)	0.023
PCI or CABG in past 30 d	10 (0.4)	9 (0.4)	0.017
Troponin level elevation in past year^a^	120 (4.4)	114 (4.5)	–0.006
Unstable angina in past year	26 (1.0)	26 (1.0)	–0.008
Severe CHF diagnosis^a^	75 (2.8)	69 (2.8)	0.001
Severe COPD diagnosis^a^	155 (5.7)	143 (5.7)	0.001
Dialysis	16 (0.6)	14 (0.6)	0.004
Poorly controlled diabetes (HbA_1c_ >9)	137 (5.1)	110 (4.4)	0.032
Gastrointestinal bleed in past 3 mo	33 (1.2)	21 (0.8)	0.037
Cancer diagnosis or treatment in past year	221 (8.2)	181 (7.2)	0.035
Dementia	80 (3.0)	95 (3.8)	–0.046
Coagulopathy	41 (1.5)	46 (1.8)	–0.025
Platelet count <100 000/μL	26 (1.0)	14 (0.6)	0.046

Abbreviations: BP, blood pressure; CABG, coronary artery bypass graft; CEA, carotid endarterectomy; DVT, deep vein thrombosis; EF, ejection fraction; GFR, glomerular filtration rate; HbA_1c_, hemoglobin A_1c_; HMO, health maintenance organization; PCI, percutaneous coronary intervention; PE, pulmonary embolism; TIA, transient ischemic attack.

^a^ Troponin level elevation was not a formal trial exclusion criterion; we used it to identify patients at higher cardiovascular risk. Severe congestive heart failure included patients with EF less than 35%; severe chronic obstructive pulmonary disease included patients with a forced expiratory volume in the first second of expiration less than 30% predicted.

### Pragmatic Sample

#### Observed Risk of Stroke and Death per Actual Treatment Received

The observed risk of stroke or death (perioperative complications) within 30 days in the CEA cohort was 2.5% (95% CI, 2.0%-3.1%) in the pragmatic sample. The observed 5-year risk of fatal or nonfatal stroke among patients with carotid stenosis in the pragmatic sample was 7.5% (95% CI, 6.5%-8.7%) in the CEA cohort and 6.9% (95% CI, 5.8%-8.1%) in the initial medical therapy cohort (Table 2). Five-year survival in the pragmatic sample was 73.3% (95% CI, 71.6%-74.9%) for the CEA cohort and 66.9% (95% CI, 65.0%-68.7%) for the initial medical therapy cohort.

**Table 2.** Five-Year Stroke Risks in the Pragmatic and Randomized Clinical Trial–like Samples

Sample	% (95% CI)			
	Perioperative complications (stroke or death risk at 30 d)	CEA cohort 5-y stroke risk	Initial medical therapy cohort 5-y stroke risk	Risk difference
**Pragmatic sample**				
Actual treatment received^a^	2.5 (2.0 to 3.1)	7.5 (6.5 to 8.7)	6.9 (5.8 to 8.1)	NA
Emulation of ACST	NA	5.6 (4.6 to 6.5)	7.8 (6.3 to 9.3)	–2.3 (–4.0 to –0.3)
After accounting for competing risks	NA	5.4 (4.7 to 6.2)	6.2 (5.7 to 9.5)	–0.8 (–2.1 to 0.5)
**RCT-like sample**				
Actual treatment received^a^	2.4 (1.8 to 3.2)	6.7 (5.6 to 8.0)	6.2 (5.1 to 7.6)	NA
Emulation of ACST	NA	5.5 (4.5 to 6.5)	7.6 (5.7 to 9.5)	–2.1 (–4.4 to –0.2)
After accounting for competing risks	NA	5.3 (4.5 to 6.2)	6.2 (4.7 to 8.1)	–0.9 (–2.9 to 0.7)

Abbreviations: ACST, Asymptomatic Carotid Surgery Trial^3^; CEA, carotid endarterectomy; NA, not applicable; RCT, randomized clinical trial.

^a^ Obtained from the unadjusted Kaplan-Meier survivor function estimates.

#### Target Trial Results

**Figure 2.**
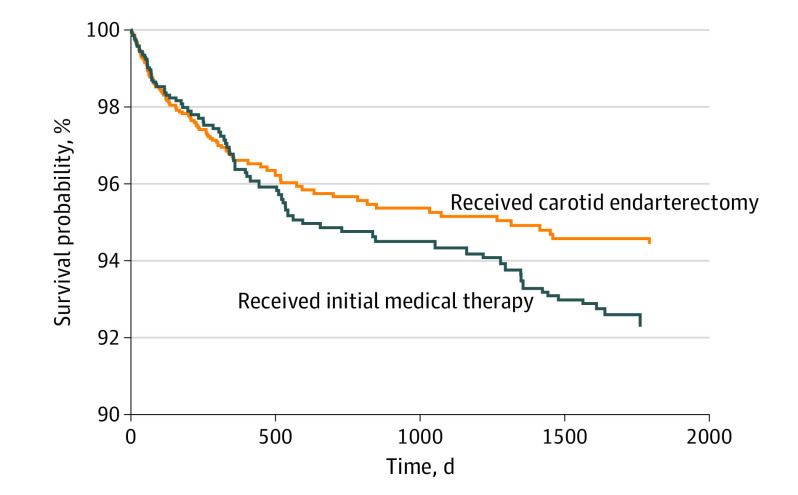
Survival Probability for Fatal and Nonfatal Strokes in the Pragmatic Sample

The baseline postrandomization characteristics for patients randomized to the 2 strategies (as part of the target trial) are provided in eTable 7 in the Supplement. After adjusting for baseline characteristics and incorporating the IPCW, the 5-year risk of fatal and nonfatal strokes was lower among patients randomized to the CEA treatment compared with those randomized to initial medical therapy (5.6% vs 7.8%; risk difference, −2.3% [95% CI, −4.0% to −0.3%]) (Figure 2, Table 2). A 5-year risk difference of 2.3% corresponded to an annualized stroke risk difference of 0.46% and a number needed to treat (NNT) of 43 over 5 years.

**Figure 3.**
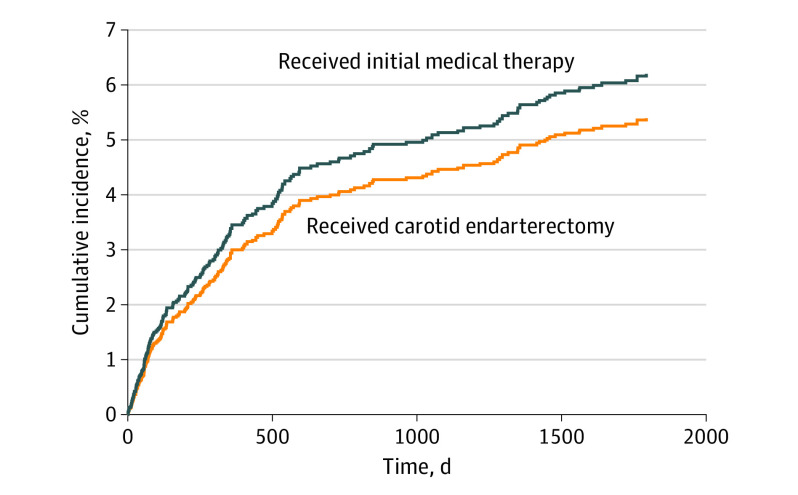
Cumulative Incidence Function of Fatal and Nonfatal Stroke in the Pragmatic Sample

The 5-year cumulative incidence function, accounting for competing risks for patients in the CEA cohort and those in the initial medical therapy cohort in the pragmatic sample, is seen in Figure 3. The stroke risk at 5 years of follow-up was 5.4% (95% CI, 4.7%-6.2%) in the CEA cohort and 6.2% (95% CI, 5.7%-9.5%) in the initial medical therapy cohort (Table 2). The risk difference between the 2 cohorts when the competing risk of death was taken into consideration was smaller than previously observed and not statistically significant at the α = .05 level (risk difference, –0.8%; 95% CI, –2.1% to 0.5%). This finding suggests that little to no difference existed between CEA and initial medical therapy when the competing risk of nonstroke death was incorporated into the analysis.

### RCT-like Sample

#### Observed Risk of Stroke and Death per Actual Treatment Received

Among the 5221 total patients in the CEA and initial medical therapy cohorts in the pragmatic sample, 1319 (25.3%) were excluded because of baseline comorbidities inconsistent with RCT inclusion criteria. Thus, the RCT-like sample comprised 3902 patients (1890 in the CEA cohort and 2012 in the initial medical therapy cohort).

The observed risk of stroke or death (perioperative complications) within 30 days in the CEA cohort was 2.4% (95% CI, 1.8%-3.2%) in the RCT-like sample (Table 2). The observed 5-year risk of fatal or nonfatal stroke among patients with carotid stenosis in the RCT-like sample was 6.7% (95% CI, 5.6%-8.0%) in the CEA cohort and was 6.2% (95% CI, 5.1%-7.6%) in the initial medical therapy cohort. In the RCT-like sample, the 5-year survival was 77.3% (95% CI, 75.4%-79.1%) in the CEA cohort and 71.9% (95% CI, 69.8%-73.9%) in the initial medical therapy cohort.

#### Target Trial Results

The baseline characteristics of the propensity-matched sample comparing the CEA cohort with the initial medical therapy cohort before and after randomization are provided in eTables 8 and 9 in the Supplement. After adjusting for baseline characteristics and incorporating the IPCW among patients who met trial inclusion criteria in the RCT-like sample, the results were similar to those observed in the pragmatic sample (Table 2). The 5-year risk of fatal and nonfatal strokes was 5.5% (95% CI, 4.5%-6.5%) among patients randomized to early CEA treatment and was 7.6% (95% CI, 5.7%-9.5%) for those who received initial medical therapy, with a risk difference of −2.1% (95% CI, −4.4% to −0.2%) (Table 2 and eFigure 1 in the Supplement). A 5-year risk difference of 2.1% corresponded to an NNT of 47.

The 5-year cumulative incidence function, accounting for competing risks among patients randomized to the CEA treatment or to the initial medical therapy in the RCT-like sample, is provided in eFigure 2 in the Supplement. The stroke risk at 5 years of follow-up was 5.3% (95% CI, 4.5%-6.2%) in the CEA cohort and 6.2% (95% CI, 4.7%-8.1%) in the initial medical therapy cohort. Similar to the pragmatic sample, the risk difference in the RCT-like sample between the 2 cohorts when the competing risk of nonstroke death was taken into consideration was not statistically significant at the α = .05 level (risk difference, −0.9%; 95% CI, −2.9% to 0.7%) (Table 2).

### Sensitivity Analysis

We examined the implication for the estimated stroke risk difference of excluding patients with either of the 2 baseline characteristics that had a standardized difference greater than 0.1 (enrollment in a health maintenance organization and history of remote stroke) (eTable 10 in the Supplement). After excluding these patients, the risk difference remained −2.2% (95% CI, −4.4% to 0.1%). We also conducted a sensitivity analysis in which we excluded anyone enrolled in a health maintenance organization at baseline and during follow-up. Again, we found minimal association with the stroke risk when comparing the CEA cohort with the initial medical therapy cohort (risk difference, −2.1%; 95% CI, −4.2% to −0.1%) (eTable 10 in the Supplement).

## Discussion

We used national VA and Medicare data to mimic a carotid trial similar in design and analysis to the ACST, the most recent published trial that compared CEA with initial medical therapy among patients with asymptomatic carotid stenosis.^3^ Similar to the ACST, the survival curves in the pragmatic sample crossed within 2 years of follow-up, and, as in the ACST, higher stroke-free survival was found among patients who received CEA. However, the fatal and nonfatal stroke risk difference at 5 years between patients who were randomized to early CEA and those randomized to initial medical therapy was 2.3% and was less than the stroke risk difference of 5.4% (any stroke and perioperative death) reported in the ACST.^3^ This risk difference corresponded to an annualized net benefit in stroke reduction of only 0.46% per year and an NNT of 43 at 5 years. In other words, 43 patients needed to be revascularized within 1 year to avoid a single fatal or nonfatal stroke within 5 years. In contrast, in the ACST, the NNT was approximately 18.^3^ Furthermore, to test the robustness of this result to the competing risk of nonstroke death, we estimated cumulative incidence functions for fatal and nonfatal strokes in the CEA and initial medical therapy cohorts, accounting for the competing risk of death associated with other causes. This analysis suggested that little to no difference existed between the 2 treatment strategies after accounting for competing causes of death. These analyses, when taken together, suggest that in real-world practice medical therapy may be an equally acceptable treatment strategy for patients with asymptomatic carotid stenosis.

We repeated the analysis in the RCT-like sample and found similar results, which was contrary to our initial expectations. We hypothesized that the benefit of CEA could be observed more readily in patients who met trial inclusion criteria because these patients may live longer, accrue more stroke reduction, and have fewer perioperative complications. The analysis confirmed that patients who met the inclusion criteria were healthier and were more likely to survive 5 years. However, the perioperative complication (stroke or death) risk observed in the CEA cohort of the RCT-like sample was not statistically significantly lower than the perioperative complication rate in the pragmatic sample and was only somewhat lower than that observed in the ACST.^3^ The decreased stroke risk in patients with carotid artery stenosis, the persistent up-front perioperative risks, and the small difference in stroke risk between the 2 treatment strategies suggest that patients treated with CEA would now require a longer time to accrue enough stroke reduction benefit to justify the up-front risks of the surgical procedure.

This study confirmed the observation that the stroke risk among patients with carotid stenosis has decreased.^15^ In the past decade, stroke has dropped from the third to the fifth leading cause of death in the US.^44^ This improvement has been attributed to several factors, including more aggressive diabetes and hypertension control and advances in medical therapy (eg, statins). The decrease in stroke risk in the initial medical therapy cohort in the present study was likely associated with more effective control of atherosclerotic risk factors in the VA. However, medical therapy could be improved in both the CEA and initial medical therapy cohorts. Over the past 2 decades, the VA has been tracking blood pressure, glycemic, and lipid targets and has achieved substantial improvements in cardiovascular risk factor control.^45^ In addition, the stroke risk estimated in the 2 cohorts preceded the publication of the SAMMPRIS (Stenting and Aggressive Medical Management for Preventing Recurrent Stroke in Intracranial Stenosis) trial, which likely focused even more attention on improving the delivery of medical care. It is possible that, with the use of high-potency statins, the stroke risk has decreased even further.^46^

The analytic method used and the 2 alternative approaches considered in this comparative effectiveness study deserve some discussion.^39,40^ The intervention (CEA) can be applied at any time after carotid imaging, and thus a successful analytical strategy accounts for and avoids immortal time bias. First, we considered the landmark analysis, in which patients would be followed up from 1 year after the first carotid image (the landmark date equals the image date plus 365 days).^47,48^ Patients for whom an event occurred between the imaging date and the landmark date would be excluded. Treatment status would have been based on what occurred between the imaging date and the landmark date. Although the landmark analysis approach would avoid immortal time bias and allow for a clearly defined exposure, it would exclude all of the early events. Consequently, the landmark analysis approach would artificially make the CEA look safer because the early perioperative stroke events would be removed. Furthermore, this approach would provide limited information to inform clinical decision-making because all information on early complications would be excluded.

Second, we considered the Cox proportional hazards regression model in the full sample, treating the intervention as a time-varying covariate and reporting the association between CEA and the outcome using a hazard ratio. Patients would have been classified in the initial medical therapy cohort until they underwent CEA, at which time they would have been classified as belonging in the CEA cohort. Although the Cox approach would avoid immortal time bias and would be analytically simple to conduct, it has a primary limitation. This approach would result in an estimation of a relative hazard ratio and would not allow the estimation of absolute treatment outcomes.

Using the target trial method, we were able to estimate the absolute reduction in the risk of the outcome and hence the NNTs or numbers needed to harm, which are important quantities for medical decision-making. In contrast, the Cox proportional hazards regression model would only allow for the estimation of relative changes in the hazard of the outcome. We have reported only absolute risks, risk differences, and NNTs, none of which can be derived from the Cox approach with time-varying covariates. Therefore, we chose the target trial method to enable the analysis to both mimic an RCT, allowing for comparisons with previously published trial results, and to account for immortal time bias and time-varying confounding between the index imaging date and the treatment randomization date.

### Strengths and Limitations

This study has several strengths. First, the study used comprehensive data for US veterans. The VA provides access to longitudinal data, Medicare data, the Corporate Data Warehouse, and the Suicide Data Repository; few comparable data sources are available to researchers. Second, the sample size was larger than the ACST, allowing us to detect smaller differences in stroke risk. Third, even though the goal of the ongoing CREST-2 (Carotid Revascularization Endarterectomy Versus Stenting 2) trial^34^ is to examine the efficacy of CEA compared with initial medical therapy in the era of modern medical therapy, the results of the present study are informative and provide insights into the risks and benefits of carotid revascularization in real-world settings in which patients may have greater perioperative complications and/or poor risk factor control compared with patients enrolled in RCTs. In community settings in which the competing risk of death is not trivial, it appears that CEA may offer no benefit.

This study has several limitations. First, although perioperative strokes were accounted for in the outcome of fatal and nonfatal strokes across 5 years of follow-up, we were unable to incorporate into the analysis the perioperative complication of death associated with CEA. Second, we used data from the Suicide Data Repository to assess fatal stroke outcomes. It is possible that, despite using standard, established methods for assessing stroke in the administrative data, we may have missed stroke deaths that were not coded.^29,38^ Third, this study involved predominantly older male veterans. As such, the findings may not be generalizable to the entire population of patients who receive carotid intervention for asymptomatic carotid stenosis. However, the results should be relevant to older men in the Medicare population, which is the cohort receiving most of the revascularizations in the US.^49^ Fourth, the implementation of the analytical strategy required censoring patients when their actual treatment became discordant with the treatment to which they were randomized per the target trial methods. This informative censoring was accounted for using model-based IPCWs, and, as with most methods used for analyzing observational studies, its success depended in part on the assumption that we have measured an adequate set of variables to account for the censoring. Although we had access to detailed clinical notes and records, it is possible that patients’ actual treatment became discordant with their randomized treatment for reasons that we were unable to capture.

## Conclusions

The absolute reduction in the risk of fatal and nonfatal strokes associated with early CEA intervention found in this study appeared to be less than half the risk difference observed in trials initiated more than 2 decades ago. This reduction was no longer statistically significant when the competing risk of nonstroke deaths were accounted for in the analysis. Given the up-front perioperative risks associated with CEA, initial medical therapy may be an equally acceptable treatment strategy for the management of patients with asymptomatic carotid stenosis.

## Notes

Supplement. **eTable 1.** Diagnostic Imaging Codes **eTable 2.** Modified Recker’s High Sensitivity Algorithm **eTable 3.** Carotid Cohort Baseline Characteristics Using Administrative Data, by Pre-Matching Groups **eTable 4.** Quality Assurance Protocol **eTable 5.** Carotid Trial Exclusions (Based on Trial Exclusions in ACST, CREST 1 and CREST 2) **eTable 6.** Variables Included in Censoring Model **eTable 7.** Baseline Cohort Characteristics: Post-Randomization Groups (Pragmatic Sample) **eTable 8.** Baseline Characteristics of the RCT-like Sample Based on Actual Treatment Received **eTable 9.** Baseline Cohort Characteristics Post-Randomization (RCT-like Sample) **eFigure 1.** 5-Year Stroke-Free Survival Among Patients Who Were Randomized to Carotid Endarterectomy Compared to Those Randomized to Initial Medical Therapy After Adjusting for Baseline Sharacteristics and Incorporating IPCW in the Analysis (RCT-like Sample) **eFigure 2.** 5-Year Cumulative Incidence of Stroke Accounting for Competing Risks Among Patients Who Were Randomized to Carotid Endarterectomy Compared to Those Randomized to Initial Medical Therapy (RCT-like Sample) **eTable 10.** Sensitivity Analyses
