# Supplementary material for: Comparative Effectiveness of Carotid Endarterectomy vs Initial Medical Therapy in Patients With Asymptomatic Carotid Stenosis
Source: JAMA Neurol. 2020 Jun 1;77(9):1–12. doi: 10.1001/jamaneurol.2020.1427 (PMC7265126; doi:10.1001/jamaneurol.2020.1427)
Supplement: Supplement. — eTable 1. Diagnostic Imaging Codes eTable 2. Modified Recker’s High Sensitivity Algorithm eTable 3. Carotid Cohort Baseline Characteristics Using Administrative Data, by Pre-Matching Groups eTable 4. Quality Assurance Protocol eTable 5. Carotid Trial Exclusions (Based on Trial Exclusions in ACST, CREST 1 and CREST 2) eTable 6. Variables Included in Censoring Model eTable 7. Baseline Cohort Characteristics: Post-Randomization Groups (Pragmatic Sample) eTable 8. Baseline Characteristics of the RCT-like Sample Based on Actual Treatment Received eTable 9. Baseline Cohort Characteristics Post-Randomization (RCT-like Sample) eFigure 1. 5-Year Stroke-Free Survival Among Patients Who Were Randomized to Carotid Endarterectomy Compared to Those Randomized to Initial Medical Therapy After Adjusting for Baseline Sharacteristics and Incorporating IPCW in the Analysis (RCT-like Sample) eFigure 2. 5-Year Cumulative Incidence of Stroke Accounting for Competing Risks Among Patients Who Were Randomized to Carotid Endarterectomy Compared to Those Randomized to Initial Medical Therapy (RCT-like Sample) eTable 10. Sensitivity Analyses [file jamaneurol-e201427-s001.pdf]

Supplementary Online Content

Keyhani S, Cheng EM, Hoggatt KJ, et al. Comparative effectiveness of carotid endarterectomy vs initial medical therapy in patients with asymptomatic carotid stenosis. *JAMA Neurol.* Published online June 1, 2020. doi:10.1001/jamaneurol.2020.1427

eTable 1. Diagnostic Imaging Codes

eTable 2. Modified Recker's High Sensitivity Algorithm

eTable 3. Carotid Cohort Baseline Characteristics Using Administrative Data, by Pre-Matching Groups

eTable 4. Quality Assurance Protocol

eTable 5. Carotid Trial Exclusions (Based on Trial Exclusions in ACST, CREST 1 and CREST 2)

eTable 6. Variables Included in Censoring Model

eTable 7. Baseline Cohort Characteristics: Post-Randomization Groups (Pragmatic Sample)

eTable 8. Baseline Characteristics of the RCT-like Sample Based on Actual Treatment Received

eTable 9. Baseline Cohort Characteristics Post-Randomization (RCT-like Sample)

eFigure 1. 5-Year Stroke-Free Survival Among Patients Who Were Randomized to Carotid Endarterectomy Compared to Those Randomized to Initial Medical Therapy After Adjusting for Baseline Sharacteristics and Incorporating IPCW in the Analysis (RCT-like Sample)

eFigure 2. 5-Year Cumulative Incidence of Stroke Accounting for Competing Risks Among Patients Who Were Randomized to Carotid Endarterectomy Compared to Those Randomized to Initial Medical Therapy (RCT-like Sample)

eTable 10. Sensitivity Analyses

This supplementary material has been provided by the authors to give readers additional information about their work.

© 2020 Keyhani S et al. *JAMA Neurology.*

eTable 1. Diagnostic Imaging Codes

**Carotid Angiogram**	**Carotid Angiogram**<br>• 75650 Angiography, cervicocerebral, catheter, including vessel origin, radiological supervision and interpretation<br>• 75660 Angiography, external carotid, unilateral, selective, radiological supervision and interpretation<br>• 75662 Angiography, external carotid, bilateral, selective, radiological supervision and interpretation<br>• 75665 Angiography, carotid, cerebral, unilateral, radiological supervision and interpretation<br>• 75671 Angiography, carotid, cerebral, bilateral, radiological supervision and interpretation<br>• 75676 Angiography, carotid, cervical, unilateral, radiological supervision and interpretation<br>• 75680 Angiography, carotid, cervical, bilateral, radiological supervision and interpretation<br>• 75860 Angiography, external carotid, unilateral, selective, radiological supervision and interpretation<br>• 88.41 Arteriography of cerebral arteries
**CT Angiography**	• 70496 Computed tomographic angiography, head with contrast material, including non contrast images and image post-processing<br>• 70498 Computed tomographic angiography, neck, with contrast material(s), including non contrast images, if performed, and image postprocessing
**Ultrasound**	• 93875 Noninvasive physiologic studies of extracranial arteries, complete bi lateral study (e.g., periorbital flow direction with arterial compression, ocular pneumoplethysmography, Doppler ultrasound spectral analysis)<br>• 93880 Duplex scan of extracranial arteries; complete bilateral study<br>• 93882 Duplex scan of extracranial arteries; unilateral or limited study<br>• 88.71 Diagnostic ultrasound of head and neck
**MR angiogram**	• 70547 Magnetic resonance angiography, neck; without contrast material(s)<br>• 70548 Magnetic resonance angiography, neck; with contrast material(s)<br>• 70549 Magnetic resonance angiography, neck; without contrast material(s), followed by contrast material(s) and further sequences<br>• 88.96 Other intraoperative magnetic resonance imaging

© 2020 Keyhani S et al. *JAMA Neurology.*

eTable 2. Modified Recker's High Sensitivity Algorithm

The original Reker algorithm was modified to include eye strokes and transient ischemic attacks (TIA). We combined VA and Medicare data to exclude any patient with evidence of stroke or TIA in the 6 months prior to the index carotid image using the following high sensitivity algorithm:
i) Stroke high sensitivity diagnosis algorithm:<br>If admission or discharge primary diagnosis:<br>• 362.31 central retinal artery occlusion<br>• 362.32 retinal arterial branch occlusion<br>• 362.33 partial retinal arterial occlusion<br>• 362.34 transient retinal arterial occlusion<br>• 430.xx, Subarachnoid hemorrhage<br>• 431.xx Intracerebral hemorrhage<br>• 432.xx Other and unspecified intracranial hemorrhage<br>• 433.01 occlusion and stenosis of basilar artery with infarction<br>• 433.11 occlusion and stenosis of carotid artery with infarction<br>• 433.21 occlusion and stenosis of vertebral artery with infarction<br>• 433.31 occlusion and stenosis of multiple and bilateral arteries with infarction<br>• 433.81 occlusion and stenosis of other specified precerebral arteries with infarction<br>• 433.91 occlusion and stenosis of other unspecified precerebral arteries with infarction<br>• 434.01, occlusion of cerebral arteries with infarction<br>• 434.11 cerebral embolism with cerebral infarction<br>• 434.91 cerebral artery occlusion, unspecified with infarction<br>• 435.x transient cerebral ischemia<br>• 436.xx Acute, but ill-defined, cerebrovascular disease<br>• 997.02 iatrogenic stroke<br><br>j) Rehab Admission or discharge primary diagnosis is V57.xx (Rehabilitation) and any secondary diagnosis;<br>• 342.xx Hemiplegia/hemiparesis<br>• 362.31 central retinal artery occlusion<br>• 362.32 retinal arterial branch occlusion<br>• 362.33 partial retinal arterial occlusion<br>• 362.34 transient retinal arterial occlusion<br>• 430.xx, Subarachnoid hemorrhage<br>• 431.xx, Intracerebral hemorrhage<br>• 432.xx, Other and unspecified intracranial hemorrhage<br>• 433.01 occlusion and stenosis of basilar artery with infarction<br>• 433.11 occlusion and stenosis of carotid artery with infarction<br>• 433.21 occlusion and stenosis of vertebral artery with infarction<br>• 433.31 occlusion and stenosis of multiple and bilateral arteries with infarction<br>• 433.81 occlusion and stenosis of other specified precerebral arteries with infarction<br>• 433.91 occlusion and stenosis of other unspecified precerebral arteries with infarction<br>• 434.01, occlusion of cerebral arteries with infarction<br>• 434.11 cerebral embolism with cerebral infarction<br>• 434.91 cerebral artery occlusion, unspecified with infarction<br>• 435.xx transient ischemic attack<br>• 436.xx, Acute, but ill-defined, cerebrovascular disease<br>• 437.xx other and ill-defined cerebrovascular disease<br>• 438.xx, or late effects of cerebrovascular disease OR<br>• 997.02 iatrogenic stroke<br><br>k) Admission or discharge primary diagnosis is 433.xx and 434.xx and any secondary diagnosis code;

© 2020 Keyhani S et al. *JAMA Neurology.*

- 342.xx, (hemiparesis)
- 362.31 central retinal artery occlusion
- 362.32 retinal arterial branch occlusion
- 362.33 partial retinal arterial occlusion
- 362.34 transient retinal arterial occlusion
- 430.xx, Subarachnoid hemorrhage
- 431.xx, Intracerebral hemorrhage
- 432.xx, Other and unspecified intracranial hemorrhage
- 433.01 occlusion and stenosis of basilar artery with infarction
- 433.11 occlusion and stenosis of carotid artery with infarction
- 433.21 occlusion and stenosis of vertebral artery with infarction
- 433.31 occlusion and stenosis of multiple and bilateral arteries with infarction
- 433.81 occlusion and stenosis of other specified precerebral arteries with infarction
- 433.91 occlusion and stenosis of other unspecified precerebral arteries with infarction
- 434.01, occlusion of cerebral arteries with infarction
- 434.11 cerebral embolism with infarction
- 434.91 cerebral artery occlusion, unspecified
- 435.xx transient cerebral ischemia
- 436.xx, Acute, but ill-defined, cerebrovascular disease
- 997.02 iatrogenic stroke

l) Anyone that got a carotid image during a hospitalization and had any TIA or Stroke code from the list above in the discharge diagnosis.

m) Stroke high sensitivity part 4: anyone that got a carotid image during a hospitalization and had any TIA or Stroke code from the list above in the discharge diagnosis.
NOTE: this is just the algorithm above but applied to those whose index carotid image happened while in the hospital

d) Exclude anyone seen in ED or urgent care (stop codes 101,130,131) setting image with clinic stop code with TIA code in 6 months prior to image date from the list below:
- 435 transient cerebral ischemia
- 362.31 central retinal artery occlusion
- 362.32 retinal arterial branch occlusion
- 362.33 partial retinal arterial occlusion
- 362.34 transient retinal arterial occlusion

e) Exclude anyone in the outpatient setting in ophthalmology or optometry clinic (stop codes 407,408) with a new code from the following list. New is defined by having the code only in the past 6 months and NOT having the code in the preceding 4.5 years.
- 435 transient cerebral ischemia
- 362.31 central retinal artery occlusion
- 362.32 retinal arterial branch occlusion
- 362.33 partial retinal arterial occlusion
- 362.34 transient retinal arterial occlusion

f) Exclude anyone with a new 435.xx code in the outpatient setting - Primary care (stop codes 322, 323, 348) internal medicine (27, 301), geriatrics (318, 319, 350), neurology (315), endocrine (305, 306), cardiology/hypertension/pulmonary (303, 309, 312). New is defined by having the code only in the past 6 months and NOT having the code in the preceding 4.5 years.

© 2020 Keyhani S et al. *JAMA Neurology.*

eTable 3. Carotid Cohort Baseline Characteristics Using Administrative Data, by Pre-Matching Groups (CAS vs. CEA vs. MT)

	CAS N = 615		CEA N = 3180		MT N = 9576	
	**N**	**%**	**N**	**%**	**N**	**%**
**Sociodemographic Characteristics**						
**Age (mean)**	74.0	6.0	73.6	6.0	76.4	6.7
**Race**						
White	569	92.5	3008	94.6	8863	92.6
Black	41	6.7	135	4.3	584	6.1
Other	5	0.8	37	1.2	129	1.4
**Ethnicity**						
Hispanic	20	3.3	114	3.6	309	3.2
Non-Hispanic	595	96.8	3066	96.4	9267	96.8
**Gender**						
Male	608	98.9	3143	98.8	9424	98.4
Female	7	1.1	37	1.2	152	1.6
**Married**	359	58.4	1839	57.8	5645	59.0
**Veteran Priority Score**						
High	532	86.5	2604	81.9	7719	80.6
Low	72	11.7	547	17.2	1737	18.1
Unknown / Missing	11	1.8	29	0.9	120	1.3
**Enrolled in Medicaid**	39	6.3	158	5.0	511	5.3
**Enrolled in Medicare HMO**	66	10.7	371	11.7	1306	13.6
**Comorbid Conditions (% yes)**						
Hypertension	567	92.2	2805	88.2	8576	89.6
Hyperlipidemia	538	87.5	2730	85.9	8192	85.6
Diabetes	261	42.4	1236	38.9	3890	40.6
Ischemic Heart Disease	373	60.7	1475	46.4	5234	54.7
Remote stroke or TIA (>6 months prior)	187	30.4	660	20.8	1902	19.9
Hemiplegia or Other Paralytic Syndrome	23	3.7	61	1.9	308	3.2
Peripheral Vascular Disease	183	29.8	768	24.2	2513	26.2
Abdominal Aortic Aneurysm	42	6.8	170	5.4	607	6.3
Atrial Fibrillation	76	12.4	312	9.8	1377	14.4
Arrythmia other than Atrial Fibrillation	76	9.1	312	5.7	1377	7.6
Valvular Heart Disease	82	13.3	234	7.4	1103	11.5
Deep Venous Thrombosis or Pulmonary Embolus	7	1.1	29	0.9	138	1.4
Hepatitis	8	1.3	42	1.3	79	0.8
Rheumatoid Arthritis	4	0.7	35	1.1	116	1.2
Pulmonary Fibrosis	3	0.5	20	0.6	93	1.0
Prostate Cancer	33	5.4	207	6.5	565	5.9
Congestive Heart Failure	344	55.9	1446	45.5	2941	30.7
Chronic Obstructive Pulmonary Disease	208	33.8	779	24.5	2544	26.6
Chronic Kidney Disease	318	51.7	1470	46.2	5302	55.4

© 2020 Keyhani S et al. *JAMA Neurology.*

**Vital Statistics (mean, SD)**						
Mean Systolic blood pressure	135.4	22.1	136.6	19.8	135.6	21.1
Mean Diastolic Blood pressure	68.9	11.8	70.2	11.5	68.5	11.8
Mean BMI	28.2	4.6	28.0	4.6	27.6	4.8
**Procedures**						
Defibrillator	18	2.9	22	0.7	159	1.7
Pacemaker	23	3.7	49	1.5	346	3.6
**Mental Health past year**						
PTSD	16	2.6	84	2.6	290	3.0
Depression	10	1.6	31	1.0	114	1.2
Anxiety	15	2.4	93	2.9	344	3.6
Psychosis	27	4.4	96	3.0	399	4.2
**Social and Behavior Risk Factors**						
Current Smoker	206	33.5	1018	32.0	2396	25.0
Alcohol Abuse past year	38	6.2	230	7.2	475	5.0
Drug Abuse past year	5	0.8	48	1.5	157	1.6
**Utilization**						
ICU visits (1 or more)	71	11.5	208	6.5	1050	11.0
Hospital admissions (1 or more)	201	32.7	680	21.4	2549	26.6
**Functional status**						
Nursing home in past year	5	0.8	22	0.7	190	2.0
**Medications**						
Antiplatelet	512	83.3	2564	80.6	6210	64.9
Antiarrhythmic	17	2.8	48	1.5	206	2.2
Steroids	57	9.3	292	9.2	940	9.8
Benzodiazepines	73	11.9	299	9.4	1158	12.1
Opioids	58	9.4	239	7.5	713	7.5
Antianginal	194	31.5	615	19.3	2450	25.6
DMARD	5	0.8	42	1.3	111	1.2
Anticoagulant	11	1.8	43	1.4	112	1.2
**Adherence**						
Adherence to antihypertension medications						
Yes	431	70.1	2144	67.4	6515	68.0
No	136	22.1	661	20.8	2061	21.5
Not hypertensive	48	7.8	375	11.8	1000	10.4
Adherence to Statins						
Yes	277	45.0	1424	44.8	4353	45.5
No	194	31.5	999	31.4	2755	28.8

© 2020 Keyhani S et al. *JAMA Neurology.*

Not on statin	144	23.4	757	23.8	2468	25.8
**Trial Exclusions**						
Acute Myocardial Infarction in past 30 days	7	1.1	29	0.9	182	1.9
PCI past 30 days/CABG past 30 days	3	0.5	13	0.4	52	0.5
Troponin elevation in past year*	51	8.3	154	4.8	797	8.3
Unstable angina in past year	10	1.6	31	1.0	150	1.6
Severe CHF diagnosis*	40	6.5	101	3.2	708	7.4
Severe COPD diagnosis*	85	13.8	199	6.3	1038	10.8
Dialysis	8	1.3	20	0.6	133	1.4
Poorly controlled diabetes (hgba1c>9)	36	5.9	157	4.9	402	4.2
GI bleed in past 3 months	4	0.7	43	1.4	210	2.2
Cancer Diagnosis or Treatment in past year	89	14.5	277	8.7	1039	10.9
Dementia	14	2.3	82	2.6	112	1.2
Coagulopathy	10	1.6	57	1.8	220	2.3
Platelet count <100k	7	1.1	23	0.7	113	1.2

*Troponin elevation is not a formal trial exclusion. It was used to identify patients at higher cardiovascular risk. Patients with a hospitalization in the past 6 months with a diagnosis of CHF or COPD were considered to have severe CHF or COPD.

© 2020 Keyhani S et al. *JAMA Neurology.*

eTable 4. Quality Assurance Protocol

A detailed training and abstraction manual was created and used for research assistant training. Each research assistant was trained in medical record review prior to initiation of data abstraction and was given 20 cases to abstract for training purposes. After initial training was completed, each research assistant was tested in a training database that was a replicate of the main abstraction data base. Each research assistant had to achieve agreement >90% against randomly selected previously abstracted cases to begin abstraction. Research assistants brought any case in which they were unsure of stenosis, symptom, or procedure status for review at weekly study meetings. Cases flagged by research assistants were reviewed by study investigators (SK, EC) and whether the patient met inclusion criteria was resolved by discussion. Patients that had ambiguous eye symptoms were also reviewed by an ophthalmologist (AN). Questions that related to type of procedure performed was also reviewed by a vascular surgeon (JJ). To ensure ongoing high-quality data abstraction 4 cases were randomly selected by the statistician each week and were independently abstracted by study investigators and compared to the research assistant abstractions at meetings with research assistants. Any differences were discussed and incorporated into training of research assistants.

© 2020 Keyhani S et al. *JAMA Neurology.*

eTable 5. Carotid Trial Exclusions (Based on Trial Exclusions in ACST, CREST 1 and CREST 2) *

	NOTES ON HOW EXCLUSIONS WERE IDENTIFIED
Heparin induced thrombocytopenia**	ICD9 codes for heparin induced thrombocytopenia**
Recent GI bleed	ICD9 codes
Severe dementia	ICD9 codes, chart review and use of medications
Symptomatic status- no stroke/TIA within 180 days of index	Reker's high sensitivity algorithm and chart review
Recent (<7 days) stroke of sufficient size (on CT or MRI) at risk for hemorrhagic conversion	Reker's high sensitivity algorithm and chart review
Spontaneous intracranial hemorrhage (past year)	ICD9 codes
Prior intracranial hemorrhage that represents risk for perioperative antiplatelet treatment	Reker's high sensitivity algorithm and chart review
Fleeting/Fixed neurologic deficit for which stroke/TIA cannot be ruled out	Reker's high sensitivity algorithm and chart review
CHF with EF < 30	Chart review
Unstable Angina	ICD9 codes
Cancer with the exception of superficial skin cancers and localized prostate cancer	ICD9 codes, receipt of chemotherapy in prior year
Respiratory Insufficiency, FEV1 <30% predicted	Chart review
Bleeding disorders (coagulopathy/active bleeding diathesis) or severe anemia	ICD9 codes
MI within previous 30 days	ICD9 codes and troponin elevations
Dialysis dependent renal failure	ICD9 codes and CPT codes
Uncontrolled diabetes	Hemoglobin A1C>9 g/dl or fasting blood sugar >400
Platelet count <100,000 µL	
Any major surgery, trauma, revascularization procedure or acute coronary syndrome (past 30 days)	ICD9 codes, CPT codes
Serum creatinine ≥ 2.5 mg/dl or the estimated GFR is < 30 cc/min.	Laboratory tests for creatinine and GFR
Extreme morbid obesity that would compromise patient safety	BMI
Major (non-carotid) surgery/procedures planned within 3 months after enrollment	Time varying covariates include valve procedures after index carotid image.
Carotid stenosis must be treatable with CEA, CAS, or either procedure	No data available
Any condition that precludes proper angiographic assessment or makes percutaneous arterial access unsafe.	No data available
Ipsilateral intracranial aneurysm >5 mm	No data available
Anatomic exclusion criteria that would preclude surgery or stenting	No data available

* we did not apply the exclusions of atrial fibrillation and cardiac source of emboli (e.g., valvular heart disease) that have been used in RCTs because these exclusions were included in trials to reduce the chance that subsequent strokes were being caused by factors other than carotid stenosis. In other words, these were exclusions designed to ensure the estimates of the efficacy of CEA was not clouded by other risk factors associated with increased stroke risk. In community practice patients with atrial fibrillation or valvular heart disease are routinely operated on. ** no one identified in sample with this condition.

© 2020 Keyhani S et al. *JAMA Neurology.*

eTable 6. Variables Included in Censoring Model

Baseline Variables Included in Censoring Model	Time Varying Variables Included in Censoring Model*
**Socio-demographics:** Age, Race, Ethnicity, Married, Veteran Priority Score, Medicaid, Medicare HMO enrollment<br><br>**Comorbid Conditions:** Hypertension, Hyperlipidemia, Diabetes, Ischemic Heart Disease, Remote stroke or TIA (>6 months prior), Hemiplegia or other Paralytic Syndrome, Peripheral Vascular Disease, Abdominal Aortic Aneurysm, Atrial Fibrillation, Arrythmia other than Atrial Fibrillation, Valvular Disease, Deep Venous Thrombosis (DVT) or Pulmonary Embolism (PE), Hepatitis, Rheumatoid Arthritis, Pulmonary Fibrosis, Prostate Cancer, Congestive Heart Failure (CHF), Chronic Obstructive Pulmonary Disease (COPD), Chronic Kidney Disease<br><br>**Vital Statistics:** Systolic blood pressure, Diastolic Blood pressure, Body Mass Index<br><br>**Procedures:** Defibrillator, Pacemaker<br><br>**Mental Health past year:** Post-Traumatic Stress Disorder, Depression, Anxiety, Psychosis<br><br>**Social and Behavior Risk Factors:** Current Smoker, Alcohol Abuse past year, Drug Abuse past year<br><br>**Utilization:** ICU visits (1 or more), Hospital admissions (1 or more) in past 6 months<br><br>**Functional status:** Nursing home in past year<br><br>**Medications:** Antiplatelet, Antiarrhythmic, Steroids, Benzodiazepines, Opioids, Antianginal, Disease-modifying antirheumatic drugs (DMARD), Anticoagulant in past year<br><br>**Adherence:** Adherence to antihypertension medications Adherence to Statins<br><br>**Trial Exclusions: Acute** Myocardial Infarction/Coronary revascularization in past 30 days, Troponin elevation past 30 days, Unstable angina in past year, Severe Congestive Heart Failure**, Severe Chronic Obstructive Pulmonary Disease** Dialysis, Poorly controlled diabetes (hgba1c>9)<br>GI bleed in past 3 months, Cancer Diagnosis or Treatment in past 2 years, Dementia, Coagulopathy, Platelet count <100k	Hospitalization or ICU visit<br>Acute Myocardial Infarction<br>Percutaneous Coronary Intervention<br>Troponin elevation (> .4, > 0.8, > 0.1)<br>Unstable angina<br>New CHF diagnosis<br>New COPD diagnosis<br>Dialysis<br>Poorly controlled diabetes (hgba1c>9)<br>GI bleed in past 3 months<br>Cancer Diagnosis or Treatment<br>Dementia<br>Coagulopathy<br>Platelet count <100k<br>Nursing Home admission in past year, DVT or PE<br>New Hemiplegia

* For post-baseline covariates, we created a binary variable that equals 1 when a patient has documentation of any of the post-baseline variables at a given point in time.

** Severe Congestive Heart Failure included patients with EF <35% or hospitalization for CHF in past 6 months, Severe COPD included patients with FEV1 of <30% predicted or hospitalization for COPD in past 6 months

© 2020 Keyhani S et al. *JAMA Neurology.*

eTable 7. Baseline Cohort Characteristics: Post-Randomization Groups (Pragmatic Sample)

	**CEA**<br><br>**N = 2886**		**MT**<br><br>**N = 2886**	
**Sociodemographic Characteristics**				
**Age (mean)**	73.7	6.0	73.7	6.0
	N	%	N	%
**Race**				
White	2749	95.3	2706	93.8
Black	112	3.9	141	4.9
Other	25	0.9	39	1.4
**Hispanic**	106	3.7	96	3.3
**Male**	2845	98.6	2855	98.9
**Married**	1685	58.4	1687	58.5
**Veteran Priority Score**				
High	2350	81.4	2322	80.5
Low	499	17.3	535	18.5
Unknown / Missing	37	1.3	29	1.0
**Enrolled in Medicaid**	142	4.9	133	4.6
**Enrolled in Medicare HMO**	342	11.9	401	13.9
**Comorbid Conditions (% yes)**				
Hypertension	2545	88.2	2585	89.6
Hyperlipidemia	2489	86.2	2529	87.6
Diabetes	1130	39.2	1144	39.6
Ischemic Heart Disease	1357	47.0	1392	48.2
Remote stroke or TIA (>6 months prior)	724	25.1	736	25.5
Hemiplegia or other Paralytic Syndrome	71	2.5	53	1.8

© 2020 Keyhani S et al. *JAMA Neurology.*

Peripheral Vascular Disease	734	25.4	711	24.6
Abdominal Aortic Aneurysm	175	6.1	149	5.2
Atrial Fibrillation	281	9.7	236	8.2
Arrythmia other than Atrial Fibrillation	179	6.2	148	5.1
Valvular Heart Disease	230	8.0	221	7.7
DVT or PE	26	0.9	23	0.8
Hepatitis	27	0.9	31	1.1
Rheumatoid Arthritis	27	0.9	36	1.3
Pulmonary Fibrosis	15	0.5	20	0.7
Prostate Cancer	141	4.9	150	5.2
Congestive Heart Failure				
Severe (EF < 35%)	129	4.5	141	4.9
Mild (EF >= 35%)	1331	46.1	1309	45.4
CHF Missing EF	36	1.3	39	1.4
No CHF	1390	48.2	1397	48.4
Chronic Obstructive Pulmonary Disease				
Moderate/Severe	270	9.4	254	8.8
Mild	235	8.1	234	8.1
Unknown Severity	239	8.3	245	8.5
No COPD	2142	74.2	2153	74.6
Chronic Kidney Disease				
Severe (GFR < 30)	169	5.9	175	6.1
Mild (GFR >= 30)	1170	40.5	1187	41.1
No CKD	1547	53.6	1524	52.8

© 2020 Keyhani S et al. *JAMA Neurology.*

**Vital Signs (mean, SD)**				
Mean Systolic blood pressure	135.9	19.4	136.8	19.9
Mean Diastolic Blood pressure	69.7	11.6	69.6	11.2
Mean BMI	28.0	4.8	27.9	4.6
**Procedures**				
Defibrillator	31	1.1	30	1.0
Pacemaker	54	1.9	41	1.4
**Mental Health past year**				
PTSD	71	2.5	73	2.5
Depression	19	0.7	29	1.0
Anxiety	84	2.9	66	2.3
Psychosis	88	3.1	87	3.0
**Social and Behavior Risk Factors**				
Current Smoker	949	32.9	923	32.0
Alcohol Abuse past year	223	7.7	201	7.0
Drug Abuse past year	37	1.3	31	1.1
**Utilization**				
ICU Visits (1 or more)	189	6.6	203	7.0
Hospital admissions (1 or more)	543	18.8	570	19.8
**Functional status**				
Nursing home in past year	23	0.8	20	0.7

© 2020 Keyhani S et al. *JAMA Neurology.*

**Medications**				
Antiplatelet	2467	85.5	2472	85.7
Antiarrhythmic	41	1.4	47	1.6
Steroids	238	8.3	253	8.8
Benzodiazepines	271	9.4	276	9.6
Opioids	193	6.7	185	6.4
Antianginal	612	21.2	621	21.5
Disease-modifying antirheumatic drugs	31	1.1	31	1.1
Anticoagulant	27	0.9	32	1.1
**Adherence**				
Adherence to antihypertension medications				
Yes	1958	67.8	1987	68.9
No	587	20.3	598	20.7
Not hypertensive	341	11.8	301	10.4
Adherence to Statins				
Yes	1343	46.5	1333	46.2
No	866	30.0	895	31.0
Not on statin	677	23.5	658	22.8
**Exclusions based on Trials**				
Acute Myocardial Infarction in past 30 days	22	0.8	26	0.9
PCI past 30 days/CABG past 30 days	13	0.5	11	0.4
Troponin elevation in past year*	146	5.1	130	4.5
Unstable angina in past year	28	1.0	34	1.2

© 2020 Keyhani S et al. *JAMA Neurology.*

Severe CHF diagnosis (EF<35%)	86	3.0	93	3.2
Severe COPD diagnosis (FEV1 <30% predicted)	189	6.6	182	6.3
Dialysis	16	0.6	19	0.7
Poorly controlled diabetes (hgba1c>9)	141	4.9	140	4.9
GI bleed in past 3 months	29	1.0	33	1.1
Cancer Diagnosis or Treatment in past year	230	8.0	250	8.7
Dementia	92	3.2	96	3.3
Coagulopathy	45	1.6	48	1.7
Platelet count <100k	22	0.8	23	0.8

Troponin elevation is not a formal trial exclusion. It was used to identify patients at higher cardiovascular risk.

© 2020 Keyhani S et al. *JAMA Neurology.*

eTable 8. Baseline Characteristics of the RCT-like Sample Based on Actual Treatment Received

	CEA N=2012		MT N=1890		Standardized Differences
	N	%	N	%	
**Age (mean)**	73.7	6.0	73.7	6.0	-0.041
**Race**					
White	1908	94.8	1783	94.34	0.022
Black	82	4.1	82	4.34	-0.013
Other	22	1.1	25	1.32	-0.021
**Hispanic**	63	3.1	65	3.44	-0.017
**Male**	1988	98.8	1867	98.78	0.002
**Married**	1575	78.3	1465	77.51	0.012
**Veteran Priority Score**	420	20.9	400	21.16	
High	17	0.8	25	1.32	0.019
Low	88	4.4	76	4.02	-0.007
Unknown / Missing	211	10.5	324	17.14	-0.046
**Enrolled in Medicaid**	259	12.1	307	14.5	0.018
**Enrolled in Medicare HMO**	211	10.5	324	17.4	-0.194
**Comorbid Conditions (% with condition)**					
Hypertension	1765	87.7	1653	87.46	0.008
Hyperlipidemia	1729	85.9	1635	86.51	-0.017
Diabetes	700	34.8	674	35.66	-0.018
Ischemic Heart Disease	849	42.2	783	41.43	0.016
Remote stroke or TIA (>6 months prior)	419	20.8	488	25.82	-0.118
Hemiplegia or Other Paralytic Syndrome	35	1.7	29	1.53	0.016
Peripheral Vascular Disease	442	22.0	433	22.91	-0.023
Abdominal Aortic Aneurysm	89	4.4	87	4.6	-0.009
Atrial Fibrillation	147	7.3	80	4.23	0.132
Arrythmia other than Atrial Fibrillation	76	3.8	75	3.97	-0.010
Valvular Heart Disease	114	5.7	86	4.55	0.051
DVT/PE	11	0.6	10	0.53	0.002
Hepatitis	16	0.8	9	0.48	0.040
Rheumatoid Arthritis	24	1.2	21	1.11	0.008
Pulmonary Fibrosis	7	0.4	8	0.42	-0.012
Prostate Cancer	107	5.3	68	3.6	0.083

© 2020 Keyhani S et al. *JAMA Neurology.*

Congestive Heart Failure					
Severe (EF < 35%)	51	2.5	59	3.12	-0.035
Mild (EF >= 35%)	813	40.4	754	39.89	0.011
Missing EF	22	1.1	15	0.79	0.031
No CHF	1126	56.0	1062	56.19	-0.005
Chronic Obstructive Lung Disease (COPD)					
Moderate/Severe	44	2.2	59	3.12	-0.058
Mild	162	8.1	131	6.93	0.043
Unknown Severity	171	8.5	151	7.99	0.019
No COPD	1635	81.3	1549	81.96	-0.018
Chronic Kidney Disease					
Severe (GFR < 30)	75	3.7	85	4.5	-0.039
Mild (GFR >= 30)	795	39.5	740	39.15	0.007
No CKD	1142	56.8	1065	56.35	0.008
**Vital Signs**					
**Systolic Blood Pressure**					
Normal (<120)	285	14.2	255	13.49	0.020
Pre-hypertension (120-139)	960	47.7	877	46.4	0.026
High Blood Pressure Stage 1 (140-159)	497	24.7	508	26.88	-0.050
High Blood Pressure Stage 2 (>=160)	260	12.9	237	12.54	0.012
Unknown	10	0.5	13	0.69	-0.025
**Diastolic Blood Pressure**					
Normal (<80)	1559	77.5	1476	78.1	-0.015
Pre-hypertension (80-89)	342	17.0	331	17.51	-0.014
High Blood Pressure Stage 1 (90-99)	87	4.3	61	3.23	0.058
High Blood Pressure Stage 2 (>=100)	14	0.7	9	0.48	0.029
Unknown	10	0.5	13	0.69	-0.025
**Body Mass Index**					
Underweight	17	0.8	13	0.69	0.018
Normal or healthy weight	474	23.6	495	26.19	-0.061
Overweight	925	46.0	819	43.33	0.053
Obese	568	28.2	531	28.1	0.003
Unknown	28	1.4	32	1.69	-0.025
**Procedures**					
Defibrillator	7	0.4	14	0.74	-0.053

© 2020 Keyhani S et al. *JAMA Neurology.*

Pacemaker	22	1.1	19	1.01	0.009
**Mental Health past year**					
PTSD	44	2.2	37	1.96	0.016
Depression	13	0.7	7	0.37	0.039
Anxiety	43	2.1	36	1.9	0.017
Psychosis	47	2.3	43	2.28	0.004
**Social and Behavior Risk Factors**					
Current Smoker	604	30.0	610	32.28	-0.049
Alcohol Abuse past year	169	8.4	141	7.46	0.035
Drug Abuse past year	17	0.8	18	0.95	-0.011
**Utilization**					
ICU visits 1 or more	77	3.8	46	2.43	0.080
Hospital admissions (1 or more)	232	11.5	163	8.62	0.097
**Functional status**					
Nursing home in past year	10	0.5	12	0.63	-0.018
**Medications**					
Antiplatelet	1709	84.9	1596	84.44	0.014
Antiarrhythmic	18	0.9	16	0.85	0.005
Steroids	130	6.5	107	5.66	0.034
Benzodiazepines	145	7.2	168	8.89	-0.062
Opioids	109	5.4	88	4.66	0.035
Antianginal	328	16.3	343	18.15	-0.049
Disease-modifying antirheumatic drugs	23	1.1	18	0.95	0.019
Anticoagulant	17	0.8	11	0.58	0.031
**Adherence**					
Adherence to antihypertension medications					
Yes	1339	66.6	1266	66.98	-0.009
No	426	21.2	387	20.48	0.017
Not hypertensive	247	12.3	237	12.54	-0.008
Adherence to Statins					
Yes	938	46.6	890	47.09	-0.009
No	582	28.9	571	30.21	-0.028
Not on statin	492	24.5	429	22.7	0.041

© 2020 Keyhani S et al. *JAMA Neurology.*

**Trial Exclusions**					
Acute Myocardial Infarction in past 30 days	0	0.0	0	0.0	0
PCI past 30 days/CABG past 30 days	0	0.0	0	0.0	0
Troponin elevation in past year	0	0.0	0	0.0	0
Unstable angina in past year	0	0.0	0	0.0	0
Severe CHF diagnosis	0	0.0	0	0.0	0
Severe COPD diagnosis	0	0.0	0	0.0	0
Dialysis	0	0.0	0	0.0	0
Poorly controlled diabetes (hgba1c>9)	0	0.0	0	0.0	0
GI bleed in past 3 months	0	0.0	0	0.0	0
Cancer Diagnosis or Treatment in past year	0	0.0	0	0.0	0
Dementia	0	0.0	0	0.0	0
Coagulopathy	0	0.0	0	0.0	0
Platelet count <100,000/ul	0	0.0	0	0.0	0

© 2020 Keyhani S et al. *JAMA Neurology.*

eTable 9. Baseline Cohort Characteristics Post-Randomization (RCT-like Sample)

	**CEA**	**N = 2134**	**MT**	**N = 2118**
	N	%	N	**%**
**Sociodemographic Characteristics**				
**Age (mean)**	73.69	5.97	73.7	5.95
**Race**				
White	2033	95.3	1984	93.7
Black	81	3.8	102	4.8
Other	20	0.9	32	1.5
**Hispanic**	78	3.7	60	2.8
**Male**	2106	98.7	2092	98.8
**Married**	1289	60.4	1262	59.6
**Veteran Priority Score**				
High	1693	79.3	1646	77.7
Low	413	19.4	451	21.3
Unknown / Missing	28	1.3	21	1.0
**Enrolled in Medicaid**	97	4.6	88	4.2
**Enrolled in Medicare HMO**	259	12.1	307	14.5
**Comorbid Conditions (% yes)**				
Hypertension	1863	87.3	1871	88.3
Hyperlipidemia	1830	85.8	1848	87.3
Diabetes	747	35.0	759	35.8
Ischemic Heart Disease	901	42.2	930	43.9
Remote stroke or TIA (>6 months prior)	515	24.1	497	23.5
Hemiplegia or other Paralytic Syndrome	42	2.0	33	1.6
Peripheral Vascular Disease	501	23.5	469	22.1
Abdominal Aortic Aneurysm	102	4.8	90	4.3
Atrial Fibrillation	148	6.9	110	5.2
Arrythmia other than Atrial Fibrillation	102	4.8	71	3.4
Cardiac Source of Emboli	120	5.6	118	5.6
DVT or PE	14	0.7	8	0.4
Hepatitis	10	0.5	19	0.9
Rheumatoid Arthritis	21	1.0	26	1.2
Pulmonary Fibrosis	8	0.4	9	0.4
Prostate Cancer	90	4.2	96	4.5
Congestive Heart Failure				
Severe (EF < 35%)	67	3.1	63	3.0
Mild (EF >= 35%)	869	40.7	873	41.2
No CHF	1181	55.3	1161	54.8
Missing EF	67	0.8	63	1.0
Chronic Obstructive Pulmonary Disease				

© 2020 Keyhani S et al. *JAMA Neurology.*

Moderate/Severe	60	2.8	54	2.6
Mild	177	8.3	153	7.2
Unknown Severity	175	8.2	184	8.7
No COPD	1722	80.7	1727	81.5
Chronic Kidney Disease				
Severe (GFR < 30)	88	4.1	86	4.1
Mild (GFR >= 30)	832	39.0	841	39.7
No CKD	1214	56.9	1191	56.2
**Vital Statistics (categorical)**				
Systolic Blood Pressure				
Normal (<120)	308	14.4	302	14.3
Pre-hypertension (120-139)	1022	47.9	958	45.2
High Blood Pressure Stage 1 (140-159)	537	25.2	567	26.8
High Blood Pressure Stage 2 (>=160)	252	11.8	282	13.3
Unknown	15	0.7	9	0.4
Diastolic Blood Pressure				
Normal (<80)	1650	77.3	1675	79.1
Pre-hypertension (80-89)	371	17.4	351	16.6
High Blood Pressure Stage 1 (90-99)	84	3.9	73	3.5
High Blood Pressure Stage 2 (>=100)	14	0.7	10	0.5
Unknown	15	0.7	9	0.4
Body Mass Index				
Underweight	23	1.1	11	0.5
Normal or healthy weight	521	24.4	533	25.2
Overweight	945	44.3	960	45.3
Obese	602	28.2	592	28.0
Unknown	43	2.0	22	1.0
**Procedures**				
Defibrillator	11	0.5	13	0.6
Pacemaker	23	1.1	25	1.2
**Mental Health past year**				
PTSD	48	2.3	42	2.0
Depression	6	0.3	17	0.8
Anxiety	46	2.2	1.89	2.3
Psychosis	51	2.4	45	2.1
**Social and Behavior Risk Factors**				
Current Smoker	681	31.9	653	30.8
Alcohol Abuse past year	181	8.5	153	7.2

© 2020 Keyhani S et al. *JAMA Neurology.*

Drug Abuse past year	19	0.9	17	0.8
**Utilization**				
ICU visits (1 or more)	70	3.3	70	3.3
Hospital admissions (1 or more)	214	10.0	238	11.2
**Functional status**				
Nursing home in past year	14	0.7	8	0.4
**Medications**				
Antiplatelet	1814	85.0	1801	85.0
Antiarrhythmic	18	0.8	21	1.0
Steroids	126	5.9	130	6.1
Benzodiazepines	165	7.7	177	8.4
Opioids	111	5.2	109	5.2
Antianginal	385	18.0	386	18.2
DMARD	23	1.1	20	0.9
Anticoagulant	11	0.5	20	0.9
**Adherence**				
Adherence to antihypertension medications				
Yes	1414	66.3	1428	67.4
No	449	21.0	443	20.9
Not hypertensive	271	12.7	247	11.7
Adherence to Statins				
Yes	1011	47.4	1333	46.4
No	612	28.7	895	30.4
Not on statin	511	24.0	658	23.2
**Trial Exclusions**				
Acute Myocardial Infarction in past 30 days	**0**	0.0	**0**	0.0
PCI past 30 days/CABG past 30 days	**0**	0.0	**0**	0.0
Troponin elevation in past year	**0**	0.0	**0**	0.0
Unstable angina in past year	**0**	0.0	**0**	0.0
Severe CHF diagnosis	**0**	0.0	**0**	0.0
Severe COPD diagnosis	**0**	0.0	**0**	0.0
Dialysis	**0**	0.0	**0**	0.0
Poorly controlled diabetes (hgba1c>9)	**0**	0.0	**0**	0.0
GI bleed in past 3 months	**0**	0.0	**0**	0.0
Cancer Diagnosis or Treatment in past year	**0**	0.0	**0**	0.0
Dementia	**0**	0.0	**0**	0.0
Coagulopathy	**0**	0.0	**0**	0.0
Platelet count <100k	**0**	0.0	**0**	0.0

© 2020 Keyhani S et al. *JAMA Neurology.*

eFigure 1. 5-Year Stroke-Free Survival Among Patients Who Were Randomized to Carotid Endarterectomy Compared to Those Randomized to Initial Medical Therapy After Adjusting for Baseline Sharacteristics and Incorporating IPCW in the Analysis (RCT-like Sample)

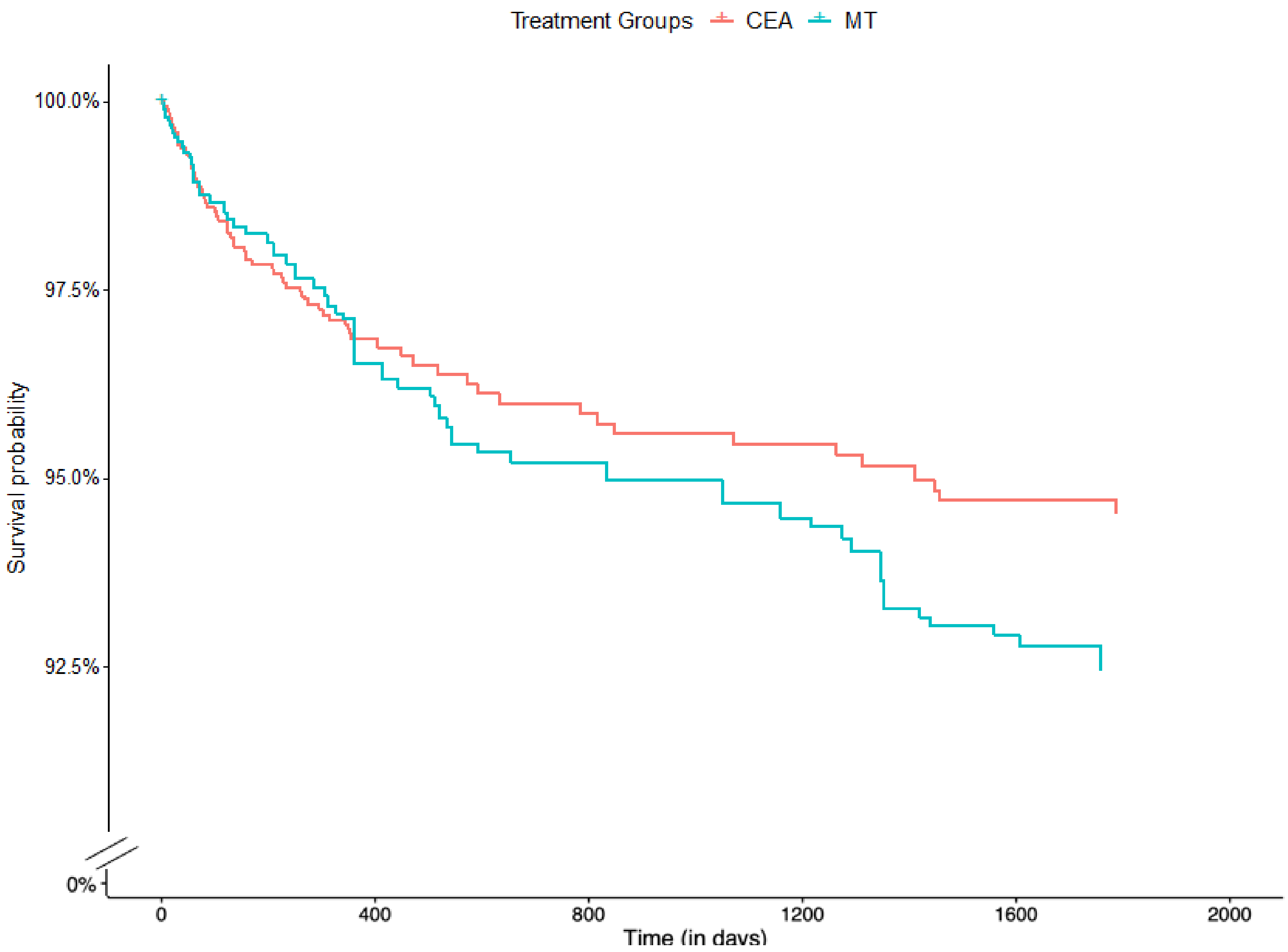

© 2020 Keyhani S et al. *JAMA Neurology.*

eFigure 2. 5-Year Cumulative Incidence of Stroke Accounting for Competing Risks Among Patients Who Were Randomized to Carotid Endarterectomy Compared to Those Randomized to Initial Medical Therapy (RCT-like Sample)

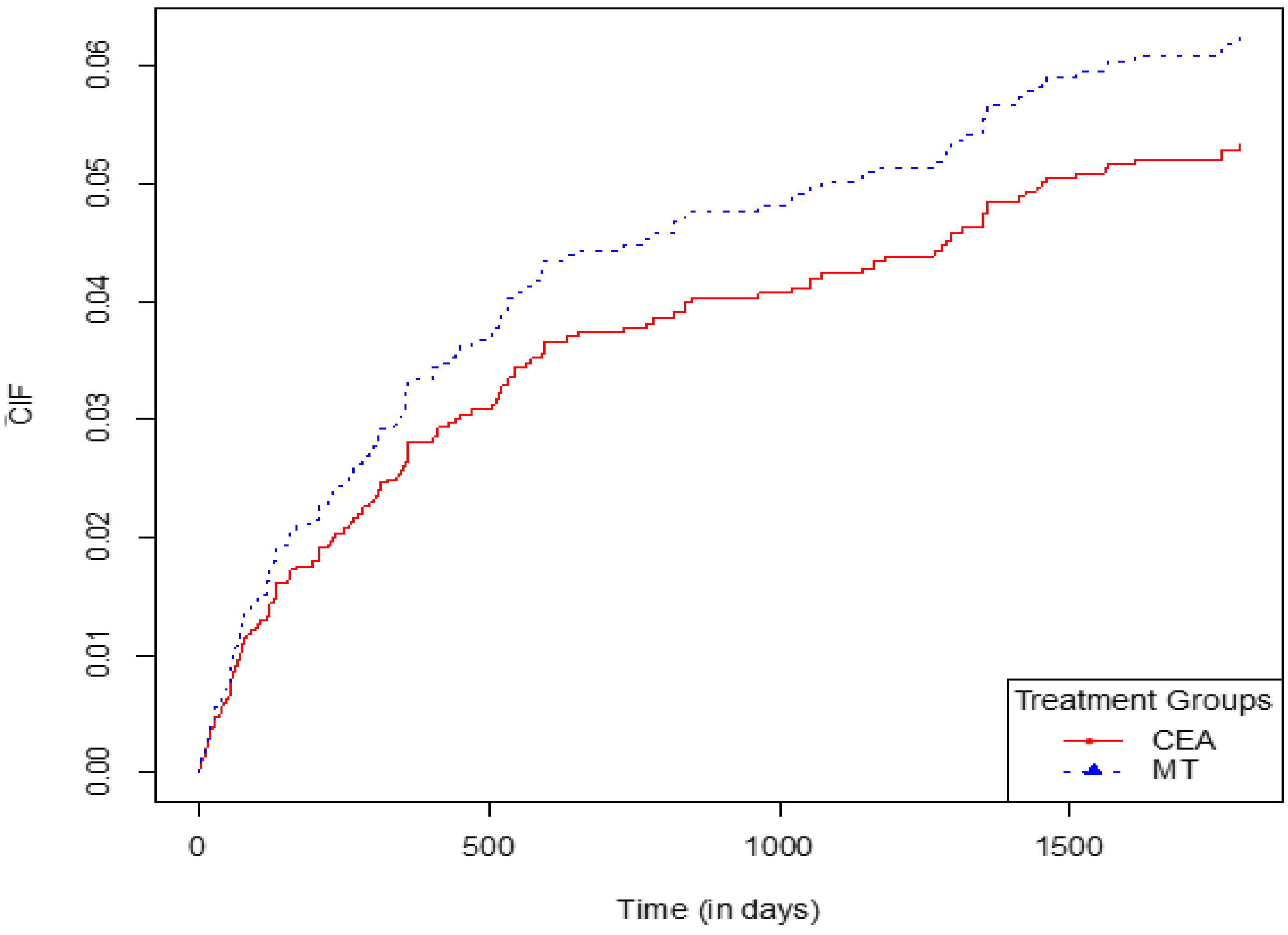

© 2020 Keyhani S et al. *JAMA Neurology.*

eTable 10. Sensitivity Analyses

Pragmatic Sample	N (sample)	CEA 5- Year Stroke Risk and 95% CI	MT 5- Year Stroke Risk and 95% CI	Risk Difference, 95% CI
Sensitivity analysis 1: excluding anyone enrolled in an HMO or who had a remote history of stroke	3736 1898 CEA; 1838 MT)	4.8% (3.5%, 6.1%)	7.0% (5.1%, 8.8%)	-2.2% (-4.4%, 0.1%)
Sensitivity analysis 2: removing anyone who entered an HMO at baseline or follow-up	4867 (2449 CEA; 2418 MT)	5.7% (4.5%, 6.9%)	7.8% (6.2%, 9.4%)	-2.1% (-4.2%, -0.1%)

© 2020 Keyhani S et al. *JAMA Neurology.*
